# Maternity Benefit in Intention and Fact

**Published:** 1913-02-01

**Authors:** 


### NATIONAL INSURANCE ACT DEPARTMENT.

### XLI.—Maternity Benefit in Intention and Fact.

We have quite recently noticed a most curious and anomalous position in regard to certain of the provisions of the National Insurance Act relating to the administration of maternity benefit. A comparison of the Bill as originally introduced into Parliament with the Act in its final shape shows that the reason for the anomaly was the acceptance of an amendment the full effect of which was not realised in the limited time allowed for discussion.

The position briefly is this. By Section 8, subsection 6, when a *married* woman who is herself an insured person is confined of a child she is entitled to sickness benefit, or disablement benefit, as the case may be, *in addition* to the maternity benefit to which *she or her husband* may be entitled. This is the principal operative section in which the conditions as to benefits are laid down, and we have indicated in italics the variation from the original Bill. The normal case contemplated by the amendment is the case where both the husband and wife are employed contributors, and it will be seen that provision is made not only for the 30s. maternity benefit, but the wife is entitled to claim sickness or disablement benefit in addition. Thus, supposing that the mother is rendered incapable of work for four weeks, and that she is entitled to sickness benefit at the rate of 7s 6d. a week, the total benefit paid in respect of the confinement would be £3. The same provision is made when the mother, being a married woman, is insured whilst the husband—for the reason, perhaps, that his income exceeds £160 a year, or that he is unable to work—is not insured.

Now the rules governing the actual administration of maternity benefit are contained in Section 18, which was not materially altered by Parliament, and the following words are quoted to make the matter quite clear: "Where the mother . . . is herself an insured person, and is not the wife . . . of an insured person, maternity benefit shall be treated as a benefit for her . . . *in any other case* the benefit shall be treated as a benefit for her husband, and shall be administered . . . by the approved society of which he is a member . . ."

Combining the effect of the two sections we have mentioned, it is seen that the maternity benefit is to come through the husband's insurance, when both husband and wife are insured, and the sickness benefit will, of course, be derived from the wife's insurance. It may be mentioned in passing that the husband and wife may be, and probably are, insured through different societies, and thus the necessity for allotting the liability for the respective payments is apparent.

Amongst the conditions entitling to benefit is the one referring to the waiting period. This period is, in the case of employed contributors, twenty-six weeks from entry into insurance, with the requirement that at least twenty-six contributions shall have been paid. In the case of voluntary contributors the period is, however, fifty-two weeks, and at least fifty-two weekly contributions must have been paid.

### An Anomaly.

We are now in a position to see the anomalous position which exists at the present time in regard to maternity benefit when the wife is insured as an employed contributor and the husband is a *voluntary* contributor. If the wife *only* had been insured as from July 15, and her contributions had been paid to date, she would have been entitled to receive from her own society, and in her own right, maternity and sickness benefits on her confinement. But because her husband is also an insured person she has no right to maternity benefit from her insurance—the 30s. maternity benefit must come from his society—and as he is a voluntary contributor no benefit can possibly accrue to him, by reason of the waiting period, until July next. Thus we have the curious situation that by entrance into insurance *voluntarily*, and without any compulsion, the husband has deprived his wife of the maternity benefit, which she would otherwise have obtained in her own right, without obtaining any corresponding advantage from his own insurance!

A somewhat similar case occurs when both the husband and wife are employed contributors and the husband's contributions are in arrear, the wife's being fully paid up to date, assuming both of them to have been insured twenty-six weeks. In this case, however, it is possible for the matter to be rectified by the payment of the arrears on the husband's insurance, and it will, of course, be to his advantage to do so. There is still a further variant, for it may have happened that the husband was out of work for several weeks after the Act came into operation, and that his entry into insurance was in consequence postponed. In these circumstances it is not possible to pay arrears, for as he was not insured no contributions can be received for the period prior to his actually becoming insured.

These instances that have been quoted show conclusively the disadvantages of hasty discussion of important legislative measures, and the acceptance of amendments which have not been considered in all their bearings.

### Another Curious Case.

Although, as we have already pointed out, maternity benefit is to be considered as a benefit for the husband when he is an insured person, it is definitely laid down in Section 19 that the husband must make adequate provision to the best of his power for the maintenance and care of his wife during her confinement and for a period of four weeks after her delivery. This is a most salutary provision, and if it is not complied with the Act empowers a sentence of one month's imprisonment to be imposed, on summary conviction, without prejudice to any other legal liabilities. The case has, however, been brought to our notice of a married man, whose wife has deserted him and is cohabiting with another. On her confinement the husband is apparently entitled to claim the maternity benefit, but if by so doing he incurs the responsibility of making provision for his wife he would, doubtless, prefer not to make the claim, and his society would be benefited accordingly. If, on the other hand, the husband does not, in the special circumstances, incur the liability, it is surely a grave public danger that he should be permitted to benefit by his wife's misconduct. The situation is fraught with many possibilities, and illustrates in a remarkable way the difficulty of making an Act of Parliament to fit every one of the complex circumstances of a nation's life.

Attendance by a Certified Midwife.

We recently pointed out that when a woman is attended by a certified midwife, and it becomes necessary for the latter to call in the services of a doctor under the rules of the Midwives Act, 1902, the doctor's fee is, subject to regulations made by the Insurance Commissioners, recoverable as part of the maternity benefit. One effect of this provision appeared to be the deferment of payment of maternity benefit when the mother had elected to be attended in the first instance by a certified midwife. The provisional regulations of the Insurance Commissioners on the matter have now been issued from which we gather that the prescribed fee is to vary according to the attendance given. Thus, for attending the mother on a summons in an emergency at any time before or within twelve hours after the end of labour the fee is 15s. For attending the mother at any subsequent time the fee is 5s., and for attending the child the fee varies according as the call is made by night (8 p.m. to 8 a.m.) or day—in the former case the fee is 5s. and in the latter 2s. 6d.

Various rules are laid down citing the cases when the fee is not recoverable from the society. Of these, perhaps, the most important is that which limits the claim on the society to ten days from the confinement, and another important rule is the one which makes it necessary for the doctor to furnish the society with a notice of the attendance in prescribed form within twenty-four hours. The maximum charge against the society is 15s., and thus the effect of the regulations is to allow a substantial payment to be made by the approved society at once, and the balance in any case need not be delayed more than ten days.

